# Fear extinction learning and anandamide: an fMRI study in healthy humans

**DOI:** 10.1038/s41398-020-01177-7

**Published:** 2021-03-15

**Authors:** Jennifer Spohrs, Martin Ulrich, Georg Grön, Michael Prost, Paul Lukas Plener, Jörg Michael Fegert, Laura Bindila, Birgit Abler

**Affiliations:** 1 Clinic for Child and Adolescent Psychiatry/Psychotherapy, Ulm University, Ulm, Germany; 2 Clinic for Psychiatry/Psychotherapy III, Ulm University, Ulm, Germany; 3 Clinic for Child and Adolescent Psychiatry, Vienna University, Vienna, Austria; 4 Department of Physiological Chemistry-Lipidomics, Mainz University, Mainz, Germany

**Keywords:** Molecular neuroscience, Psychiatric disorders, Learning and memory, Predictive markers

## Abstract

Anxiety- and trauma-related disorders are severe illnesses with high prevalence. Current treatment options leave room for improvement and the endocannabinoid system (ECS) has become a key target in psychopharmacological research. Rodent models suggest an anxiolytic effect of endocannabinoids and demonstrated that the ECS is involved in the modulation of fear learning and aversive memory consolidation. So far, one prominent target was inhibition of fatty acid amino hydrolase (FAAH), the degrading enzyme of the endocannabinoid anandamide (AEA). Research in humans remains scarce, but genetic studies have found that the single-nucleotide polymorphism (SNP) *FAAH* C385A (rs324420) is associated with lower catabolic performance of FAAH and increased levels of AEA. Translational research on the ECS in fear learning processes is rare, yet crucial to understand the mechanisms involved. To address this lack of research, we designed a fear conditioning, extinction learning paradigm with 51 healthy, male humans who underwent functional magnetic resonance imaging (fMRI) before analysing baseline and task-related changes of AEA, as well as the *FAAH* polymorphism (rs324420). The results indicate higher AEA levels in AC-heterozygotes than in CC-individuals (SNP rs324420), but no difference between the groups during extinction learning. However, neural activation of the anterior cingulate cortex and anterior insular cortex during extinction learning correlated positively with AEA baseline levels, and task-related changes in AEA were found particularly during fear extinction, with a modulatory effect on neural activation related to extinction learning. Results indicate a putative role for AEA in fear extinction learning. Pre-treatment with AEA-enhancing drugs could promote extinction learning during psychotherapeutic interventions.

## Introduction

Fear and anxiety disorders are among the most frequently diagnosed psychiatric conditions worldwide^1^. For patients, the resulting disabilities and loss in quality of life are significant and represent a serious burden to their familial environments. In order to expedite the development of more effective therapies, major research efforts are currently directed towards elucidating the underlying mechanisms of fear and anxiety disorders as well as their behavioural correlates. Particularly the neuromodulatory, genetical, physiological and behavioural processes involved in the acquisition and extinction of fear and anxiety have been intensively studied in experimental animal and human setups in the past decades, and have led to major progress in understanding underlying neurobiological processes suited to promote the development of new therapeutic approaches^2–4^.

The most commonly applied paradigm to investigate the mechanisms of fear learning stems from Pavlovian fear conditioning^5–8^. Here, an initial, neutral stimulus (e.g. an image) is coupled with an aversive stimulus (unconditioned stimulus, UCS). Over time, the neutral stimulus becomes a conditioned stimulus (CS^+^), and is able to elicit a fear response, even when presented without the UCS. For extinction learning, the CS^+^ is later on presented without the UCS, resulting in a decline in the elicited fear response^9^. Pavlovian conditioning and extinction paradigms in animals and humans have been successfully applied to enhance the understanding of anxiety disorders and to improve the development of therapeutic approaches.

Current gold standard treatments for anxiety disorders, such as cognitive behavioural therapy (CBT) have been shown to be highly effective, however, limitations in the applicability in terms of efficacy and effectiveness still leave room for improvement^10^. Therefore, research is carried out to further enhance treatments. The endocannabinoid system (ECS) with its well-known, excellent pharmacological accessibility has become one of the key targets in basic anxiety research. Evidence mainly from animal research^11,12^ suggests an important neuromodulatory role of the endocannabinoids, particularly anandamide (AEA), which is degraded by fatty acid amino hydrolase (FAAH). More precisely, augmenting AEA may enhance fear extinction^13,14^ and thus represents a candidate to acutely improve effects of psychotherapeutic interventions. Furthermore, Mayo et al.^13^ demonstrated that FAAH-inhibition increased AEA levels and enhanced extinction recall in healthy humans.

In rodent models, an increase of AEA by genetic deletion, inhibition of FAAH, or via the administration of cannabinoid receptor 1 (CB1) agonists, had a preventive function against the anxiogenic impact of aversive stimuli^15,16^. It also decreases anxiety-like behaviour by protecting from stress-induced reductions of AEA, and thus promotes fear extinction^3,14,17–23^. Conversely, genetic deletion or administration of CB1-antagonists was associated with impaired extinction learning^24^ and anxiogenic effects^25^ in animal models.

In the past years, the focus has shifted towards translational research in humans, but the translation of findings from animal models to humans, which is necessary to develop pharmacological tools, remains tentative^26–28^. The single-nucleotide polymorphism rs324420 in the *FAAH*-coding gene has been demonstrated to modulate fear extinction learning in rodent models and humans. A-allele homozygote humans demonstrate reduced FAAH activity, present higher AEA levels, and show enhanced stress-coping, greater fear extinction, and an augmented extinction recall^14,17,29,30^. However, little is known about task-related changes of AEA, and only two studies so far could demonstrate that AEA levels in healthy subjects were increased after a stress task^17,31^.

Regarding cerebral networks involved, correlates of fear conditioning and extinction in humans have been summarised by various neuroimaging studies and meta-analyses, suggesting that among others, the dorsal anterior cingulate cortex (dACC) and anterior insular cortex (AIC) are responsive in both processes^32–34^. In a first attempt to characterise effects of cannabinoids on the networks involved, Rabinak et al.^35,36^ have demonstrated that ∆9-tetrahydrocannabinol (THC) application before the extinction session enhanced extinction, extinction recall, and healthy volunteers showed heightened ventromedial prefrontal cortex (PFC) and hippocampus activation as neural signatures associated with the extinction process. Furthermore, Andrade et al.^37^ highlighted the role of the anterior insula in anxiety disorders and the subjective ‘high’, which is commonly associated with THC administration^38,39^.

In this context, the present study was designed to investigate the role of peripheral AEA in humans with respect to neural correlates of fear learning processes. For this purpose, we implemented an experimental Pavlovian-like conditioning and extinction paradigm for use in combination with functional magnetic resonance imaging (fMRI) in 55 healthy volunteers. The experimental setup was guided by previous studies to reach comparability with recent meta-analyses^33,34^. We predicted that the sample’s brain activation associated with fear extinction would replicate meta-analytical findings (1). In a second step (2), the main goal of the study was to investigate the relation of peripheral baseline AEA levels and the neural correlates of extinction learning. Based on experiments suggesting a link between peripheral and central AEA levels^17,29^, we expected neural activation associated with extinction learning to vary as a function of individual, peripheral baseline levels of AEA. Finally (3), we hypothesised that task-related changes in individual plasma levels of circulating AEA measured before and after the fear extinction task would correlate with the neural activation related to extinction learning.

## Materials and methods

### Participants

Analyses were conducted on data from 51 right-handed, male participants (mean age = 22.8 years, SD = 3.0 years). Subjects were included after completion of a screening procedure to exclude confounding medical or psychiatric conditions and substance use (Supplementary Material). All participants signed an informed consent prior to the study as approved by the Ethics Board of Ulm University, Germany.

### Experimental task during fMRI

**Fig. 1:**
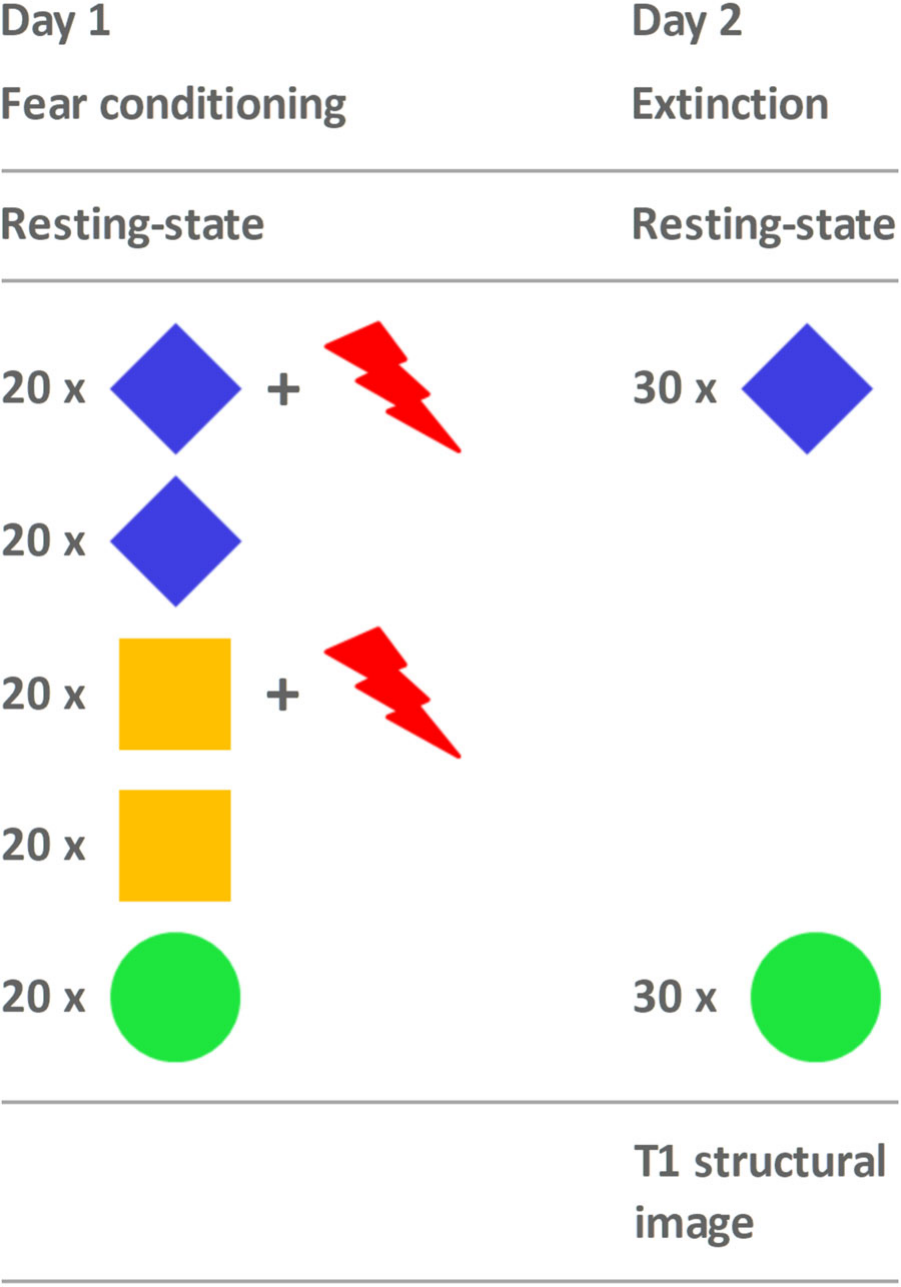
Schematic overview of the experimental setup. On day 1, 20 stimuli of each condition (CS^−^, CS^+a^, CS^+b^, CS^+a^ + US, CS^+b^ + US) were presented 20 times in pseudo-randomised order at a reinforcement rate of 50% in the case of CS^+^. Blue and yellow depict the conditioned stimuli (CS^+a^ and CS^+b^), green the neutral stimulus (CS^−^), and the red arrows represent the unpleasant, unconditioned stimulus (heat, US) applied with a thermal stimulation device on the right shinbone. To avoid biasing effects of colour or shape, shapes and colours of stimuli were balanced over subjects. For fear extinction, CS^−^ and CS^+a^ were presented 30 times each, without presentation of the US. Additionally, a T1 structural image was recorded afterwards on day 2; on both days, resting-state fMRI was acquired prior to task-based fMRI. To ease figure reading, the day 3 scenario (extinction recall) is not presented here and will be presented elsewhere.

Based on previous experiments^6,40,41^, participants underwent three phases of a Pavlovian conditioning paradigm over the course of three consecutive days: fear conditioning (day 1), fear extinction (day 2), and extinction recall (day 3) (Fig. 1). Start of the experiment was standardised between 7 AM and 9 AM. For fear conditioning, three different geometric stimuli were presented on a computer screen, with a duration of 4 s and an inter-trial interval of 8–18 s (mean = 9.9 s, SD = 2.4), during which a fixation cross was presented. Stimulus onsets were jittered by randomly adding fractions of the fMRI repetition time. One stimulus remained neutral (CS^−^) and two stimuli (CS^+a^, CS^+b^) were coupled with an individually calibrated unpleasant thermal stimulation (US) to the right shinbone (mean temperature: 46.59 °C, SD = 2.96 °C), which was applied by an fMRI-compatible ATS-thermode (Supplementary Material). On all experimental days, participants were informed that thermal stimulation was possible and the thermal stimulation device, set to a baseline temperature of 32 °C, was applied. A description of the processes of extinction recall (day 3) is beyond the scope of this paper.

### Measurement of anandamide

To investigate influences of the experiment on AEA levels, blood samples (2 × 2.7 ml EDTA) were taken, within 5 min before and 5 min after the fMRI scan on each experimental day. To further study the link between AEA levels and the *FAAH* polymorphism rs324420 (C385A)^17,29^, a separate blood sample (2 × 7.5 ml EDTA) was taken with the first blood collection (Supplementary Material). Blood to plasma processing, as well as extraction and analysis of plasma anandamide were carried out following previously described protocols^42^ and is described in more detail in the Supplements. Levels of 2-Arachidonoylglycerol were assessed as well and will be reported elsewhere.

### Anxiety ratings

Utilising a visual analogue scale and an MRI-compatible trackball (NAtA TECHNOLOGIES, Coquitlam, Canada), participants were asked to rate the subjectively perceived fear related to each stimulus^43^ before and after the fMRI experiment *(How afraid are you of the stimulus coupled to this symbol?)*. The scale ends were defined as *0 not at all – 10 very much*.

### MRI data acquisition

Acquisition of magnetic resonance imaging data was performed on a 3 Tesla MAGNETOM Prisma (Siemens AG, Erlangen, Germany) with a 64-channel head/neck coil. For estimation of task-related brain activation, the T2*-weighted BOLD signal was measured using an echo-planar imaging (EPI) pulse sequence (Supplementary Material). During the fMRI session on day 1, 705 EPI volumes were acquired, corresponding to an experimental time of 23.38 min (14.32 min or 432 EPI volumes on days 2 and 3, respectively). On day 2, after the experimental task, a high resolution T1-weighted structural image was acquired (Supplementary Material).

### Analysis of fMRI data

Image pre-processing and statistical analyses were performed using SPM12 (see Supplementary Material for details). Based on previous experiments in mice^44^, the process of extinction learning (day 2) was expected to modulate the amplitude of the BOLD responses elicited by CS^+^ and CS^−^ trials during the course of the experiment. Specifically, the extinction signal was predicted to follow an exponential decay function, and a model with parametric modulation of regressors for CS^+a^ and CS^−^ was implemented (Supplementary Material). Contrast images representing neural activation during fear extinction, and contrast images representing conditions CS^+^ and CS^−^ during fear conditioning were subjected to a random-effects analysis, implemented in SPM12 as flexible factorial design with factors Subject, Group (see Results, hypothesis 2) and Condition. The factor Group was the necessary result of exploratory analyses, where it was observed that increases and decreases of AEA levels on day 2 were equally distributed across the entire sample. Subsequently, to investigate the relation of AEA level changes and neural signalling, the entire group was divided into 4 subgroups according to quartiles in AEA post-to-pre level changes (quartile 1: high increase, quartile 2: low increase, quartile 3: low decrease, quartile 4: high decrease). Significant effects related to fear extinction learning were assessed by investigating the regressor representing the exponential signal decay for CS^+^.

After evaluating the validity of the present experiment based on the categorical analyses (hypothesis 1), the two remaining hypotheses were addressed, i.e. hypothesis 2, predicting that individual baseline AEA levels, and hypothesis 3, predicting that task-related changes in AEA would correlate with neural signals associated with fear extinction learning. For this purpose, the contrast images representing the exponentially decaying signal for CS^+a^ were used as a dependent variable within regression analyses with baseline levels of AEA (day 1) and pre-to-post changes of AEA levels on day 2 as predictors. In an exploratory manner, linear contrasts in line with the quartiles model of AEA level changes were computed.

Results of the analyses were assessed at whole-brain level by applying a threshold of *p* < 0.001 at the voxel, and FWE-corrected (*p* < 0.05) cluster-level inference, corresponding to a cluster size of at least 193 (hypothesis 1 and 3) contiguously significant voxels. For the regression analysis (hypothesis 2), a threshold of *p* < 0.001 at the voxel- and *p* < 0.05 at the cluster-level was used with a cluster size of at least 144 contiguously significant voxels.

## Results

### FAAH and anandamide levels

*FAAH* genotyping revealed 17 AC- and 34 CC allele carriers. Mean baseline plasma levels (day 1) of AEA were significantly (*t*(49) = 2.81, *p* = 0.007) higher in A-allele carriers ([AEA]_AC_ = 0.49 ± 0.16 pmol/ml) compared to the individuals homozygous for the C allele ([AEA]_CC_ = 0.38 ± 0.13 pmol/ml).

### Neuroimaging data

#### Categorical effects of fear extinction (hypothesis 1)

**Fig. 2:**
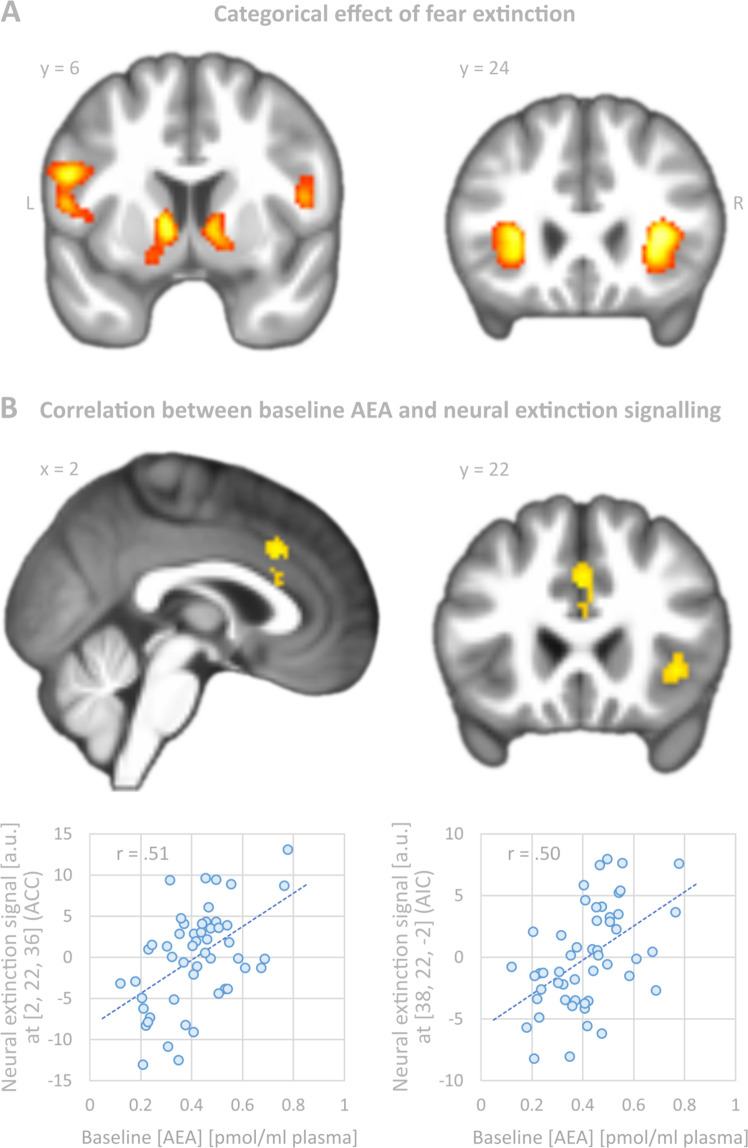
Categorical effect of fear extinction and correlation between baseline anandamide (AEA) and neural extinction signalling. **A** Brain regions with significant effects of fear extinction learning modelled as an exponentially decaying signal. Significance (whole-brain analysis) was assessed at *p* < 0.001 (voxel level) and *p* < 0.05 (cluster level, FWE-corrected, corresponding to at least 193 voxels). The statistical parametric map was superimposed on coronal and sagittal sections of the group averaged T1 image. For a statistically reliable inference of neural extinction learning, please see the summary tabulated in Table 1. **B** Whole-brain regression analysis: anandamide (AEA) baseline levels correlated positively with the degree of extinction learning on day 2 in the dorsal anterior cingulate cortex (dACC) and right anterior insula (AI). Significance was assessed at *p* < 0.001 at voxel level and *p* < 0.05 at cluster level resulting in at least 144 continuous voxels. Scatter plots refer to the voxel with the highest correlation coefficient. For demonstration purposes, fMRI sectional views were extracted from more conservative analyses with smaller cluster sizes. Coordinates refer to MNI space. L left, R right.

In line with a recent meta-analytic report^33^, neural activation in a network including the bilateral anterior insula, ventral striatum and thalamus was significantly linked to extinction learning (Table 1 and Fig. 2A), defined as an exponential decay, which highly overlapped with the network engaged during fear conditioning (see Supplementary Material and Supplementary Table 1). These regions were reliably associated with fear learning^34^ and fear extinction learning^33^ and provide support for the validity of the experimental setup.

**Table 1:** Brain regions bearing a significant effect of fear extinction at a threshold of *p* < 0.001 (voxel level) and FWE-corrected (*p* < 0.05) cluster sizes (corresponding to 193 voxels).

	Brain region	Number of voxels	Peak voxel (MNI space)			
			*X*	*Y*	*z*	*z*-score
R	Anterior insula	687	32	26	6	6.63
R	Precentral gyrus/Rolandic operculum		50	10	14	4.62
L	Anterior insula	1009	−32	28	6	6.47
L	Precentral gyrus/Rolandic operculum		−52	6	24	5.57
L	Ventral striatum	193	−10	4	0	6.11
R	Ventral striatum	298	12	8	−2	5.39
	Thalamus		0	−6	6	5.33

*L* left, *R* right.

#### Anandamide levels and fear extinction learning (hypothesis 2)

Neural extinction learning signal did not significantly differ between *FAAH* genotypes (2-samples *t*-test in SPM12; *p* > 0.025, uncorrected). Next, baseline AEA levels correlated with the degree of extinction learning (whole brain analysis) in two clusters of a cingulo-opercular network, the dACC ([0, 22, 30], *Z* = 3.81, *k* = 162) and the right AI ([38, 22, –2], *Z* = 3.74, *k* = 144) (Fig. 2B). In these regions, higher baseline AEA levels predicted stronger neural extinction learning, while pre-to-post changes in AEA levels were not significantly correlated with the neural extinction signal.

**Fig. 3:**
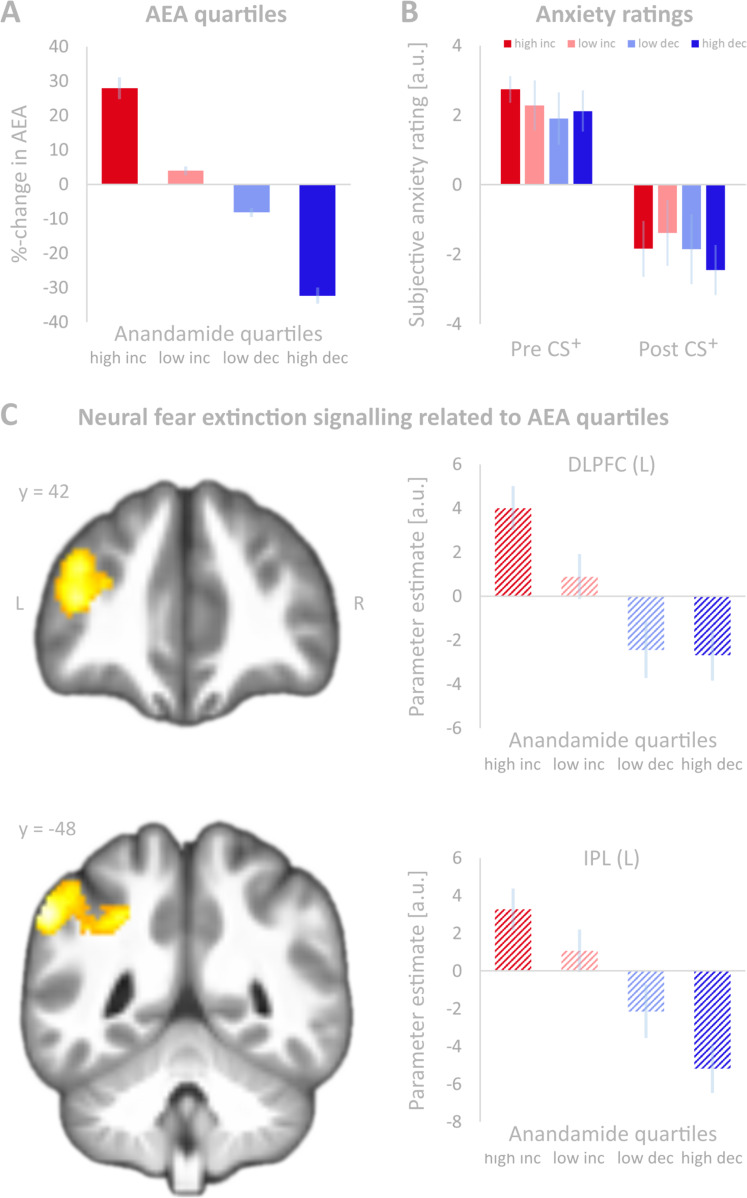
Anandamide (AEA) quartiles, anxiety ratings and neural fear extinction signalling related to AEA quartiles. **A** Relative changes (in per cent, %) in anandamide (AEA) plasma levels before and after fear extinction (day 2) grouped in quartiles (quartile 1: high increase (*n* = 13), quartile 2: low increase (*n* = 13), quartile 3: low decrease (*n* = 12), quartile 4: high decrease (*n* = 13)). Error bars denote standard errors of the mean. **B** Average subjective anxiety ratings before (pre) and after (post) fear extinction (day 2) for the CS^+^ grouped according to changes in AEA (quartiles 1–4). A rating of 0 indicates no feeling of fear in association with the stimulus, 10 indicates the greatest fear. Error bars denote standard errors of the mean. **C** Group-wise (AEA quartile model) comparison of cerebral networks showing an exponential decay during fear extinction learning. Significance was assessed at *p* < 0.001 (voxel level) and *p* < 0.05 (FWE-corrected) at the cluster-level (corresponding to 169 voxels). The statistical parametric map was overlaid on sagittal sections of the group averaged T1 image. For demonstration purposes, fMRI sectional views were extracted from more conservative analyses with smaller cluster sizes. Coordinates refer to MNI space. MFG middle frontal gyrus, IPL inferior parietal lobule, INC increase in AEA during fear extinction, DEC decrease in AEA during fear extinction.

Absence of the latter effect stipulated an exploratory approach applying the basic idea that individual AEA levels might change due to experimental fear extinction, and therefore increase or decrease relative to baseline. Therefore, individual pre-to-post changes of AEA levels on day 2 were computed, and these differences were used to divide the entire sample into four subgroups according to quartiles in AEA level changes, which occurred independently of *FAAH* genotype (Fig. 3A). Accordingly, this new factor group with four levels was implemented in a one-way ANOVA (flexible factorial design) with the degree of neural extinction learning as the dependent variable. When testing for significant parametric effects of factor group by means of *t*-contrasts, this analysis revealed that with increasing AEA levels neural activation decayed exponentially in a left-lateralized bilateral frontoparietal network, while the opposite was observed with decreasing AEA levels. The network comprised the inferior frontal gyrus, dorsolateral prefrontal cortex (DLPFC) and inferior parietal lobule (IPL) together with midline structures, i.e. precuneus and dACC (Fig. 3C and Table 2), a network, which have been linked to flexible coordination of cognitive control^45^.

**Table 2:** Brain regions bearing a significant parametric effect of fear extinction as presented by an exponential decay related to relative increases in AEA (INC groups) versus decreases (DEC groups), at a threshold of *p* < 0.001 (voxel level) and FWE-corrected (*p* < 0.05) cluster sizes (corresponding to 169 voxels).

	Brain region	Number of voxels	Peak voxel (MNI space)			
			x	y	z	z-score
L	Inferior frontal gyrus (triangular part)	1568	−36	38	14	6.35
L	Inferior frontal gyrus (opercular part)		−42	12	16	6.09
L	Dorsolateral prefrontal cortex		−36	46	22	5.34
L	Dorsal anterior cingulate cortex		−8	24	38	4.09
R	Inferior frontal gyrus (triangular part)	264	50	38	14	4.64
R	Dorsolateral prefrontal cortex		36	54	14	3.97
R	Dorsolateral prefrontal cortex	620	36	28	36	4.84
L	Precuneus	821	−6	−64	52	4.32
L	Inferior parietal lobule	975	−56	−44	44	5.12
R	Inferior parietal lobule	223	52	−38	50	3.66
R	Supramarginal gyrus		54	−42	30	3.76
L	Middle temporal gyrus	284	−54	−48	−2	4.63
L	Cerebellum	246	−36	−50	−26	4.61

*L* left, *R* right.

### Anxiety ratings

Subjective anxiety ratings related to stimuli after fear conditioning for CS^+^ (mean = 7.54; SD = 0.25) were significantly higher than for CS^−^ (mean = 1.24; SD = 0.22; *t*(50) = 18.33; *p* < 0.001). Differences remained significant before extinction learning (day 2, *t*(50) = 16.31, *p* < 0.001). Consistent with successful extinction learning, anxiety ratings decreased significantly from pre (pre CS^+^: mean = 7.26; SD = 0.31) to post-experiment (post CS^+^: mean = 4.88; SD = 0.43; *t*(50) = 10.37, *p* < 0.001). For data on anxiety ratings on day 1 (please see Supplementary Material and Supplemental Fig. 1). Considering the quartiles model, both groups displaying increases in AEA upon the fear extinction learning task, showed higher anxiety ratings before and after the experiment, while the two groups with decreases in AEA displayed lower anxiety ratings (Fig. 3B). However, this effect was not significant.

## Discussion

Based on a translational approach, we set up a fear conditioning-extinction paradigm to address a research question imposed from previous studies in rodents suggesting a core role of AEA in extinction learning. After identifying central neural structures, previously described in neuroimaging studies related to fear conditioning and extinction^37,38^, we were able to show that individual baseline levels of AEA in peripheral plasma before the experiment predicted brain activation associated with extinction learning in the anterior insular cortex (AIC) and the dorsal anterior cingulate cortex (dACC). Post extinction AEA levels were correlated with subjective ratings of anxiety related to the extinguished stimulus, corroborating a link between AEA levels and fear extinction learning, which is further supported by the results from the quartiles model. Here, results demonstrated that pre-to-post increases in AEA were associated with exponential decreases in neural activation during fear extinction in frontoparietal and midline networks of brain regions, which have previously been linked to cognitive control^45–47^. Pre-to-post decreases in AEA levels were associated with an inverse pattern.

### Relation between individual anandamide levels and fear extinction learning

Across participants, higher baseline AEA levels were associated with greater neural activation during extinction learning. This result supports the notion of AEA as a putative modulator of extinction learning as shown in previous rodent studies^3,29,48^. Furthermore, it could be interpreted in line with hypotheses suggesting that extinction learning may be enhanced by increasing AEA levels via CB1 agonism or FAAH inhibition as proposed by human studies^13,36^. With the correlation of neural extinction signalling between baseline AEA and dACC and AIC activation, we found effects in a network, which has been found to have a key role in fear extinction learning and fear processing in neuroimaging studies^33,34,37,40,49,50^. Previous pain and fear conditioning studies have suggested that the cingulo-opercular network of ACC and AI integrates nociceptive input and memory consolidation to allow the organism to prepare for future adverse stimuli, to respond appropriately^51^ and to be involved in anticipatory anxiety^52–57^. Especially the dACC has been shown to process sympathetic autonomic arousal—a key physiological component of anxiety^58,59^—and to be one of the main structures in extinction learning^60^. Thus, the attenuation in dACC and AIC activation over the course of extinction learning could reflect a reduction of fear-related arousal, which is a component of successful therapy of anxiety disorders^58,61,62^.

To our knowledge, this is the first study that has investigated changes in peripheral AEA signalling in humans before and after fear extinction learning. The observation of task-related changes in AEA levels is in line with previous studies that have pointed out a role of the ECS in down-regulating activation of the HPA-axis due to stress^63^. Previous studies suggested that the ECS may be up-regulated by acute stressors, while recovery goes along with a drop in AEA levels^13,18^. However, our data suggest that peripheral AEA is increased or decreased according to individual demands (and baseline levels), and is potentially related to neural signalling of extinction learning in a frontoparietal cognitive control network. While the overall (non-AEA-level modulated) signal during extinction learning was evident in parts of a cingulo-opercular network linked to salience and arousal processing^64^ (as expected from previous literature^32,33^), differential activation depending on AEA mobilisation was additionally evident in brain regions previously related to cognitive control. As summarised by Marek & Dosenbach^45^, neuroimaging studies suggest that frontoparietal and cingulo-opercular networks represent cognitive control. Particularly the DLPFC and IPL support performance feedback^65^ while anterolateral prefrontal regions were related to the maintenance of control^66^. Thus, the frontoparietal network was advocated to be essential for the flexible coordination of cognitive control^45^. In subjects with increasing AEA levels upon extinction learning, activation of the frontoparietal and cingulo-opercular networks followed the model of an exponential decay in neural activation. In subjects with decreases in AEA, this response pattern was reversed, suggesting that differential activity of AEA signalling may be associated with differential frontoparietal activation related to cognitive control coordination, and possibly resulting in different shifts regarding the recovery stage of the AEA levels. Cole et al.^67^ proposed that this frontoparietal and cingulo-opercular control system may have a crucial role in the maintenance of mental health and that psychiatric disorders may lead to a disruption of the functionality of the control system, resulting in limited capacities to regulate disorder-related domains. However, whether responsivity in cognitive control-related networks triggers AEA level changes or represents a consequence, remains subject of further research.

### Limitations

In rodents, particularly the basolateral amygdala and the medial PFC were identified as further functionally relevant regions in fear processing^49,68^. Similarly, in PTSD patients, neuroimaging studies revealed hyperresponsivity of the amygdala and the hippocampi together with a hyporesponsive medial PFC^69^ as an important neural feature. Both regions were absent in present statistical analyses given actual thresholds. Additionally, Fullana et al.^33^ have suggested that activation of specific brain regions, might only occur during intense states of fear (e.g. a traumatic event). Fear extinction studies apply only mild forms of fear and usually recruit healthy samples, which might not be fully suited to investigate the interaction of the ECS and fear processing networks relevant for clinical settings. Therefore, although our results seem to be well suited to extend knowledge on the interaction of the ECS and extinction learning processes, a transfer to clinical settings is still limited. Another limitation of this study is that only males were included, and the issue investigated here is certainly to be addressed in female participants. Previous studies have pointed out differences between sexes with regard to their responses to cannabis exposure^70,71^^,^^72^. Additionally, Neumeister et al.^73^ found that women displayed lower levels of AEA than men.

### Future directions

Various studies have outlined the potentially beneficial effects of ECS modulation in conditioning studies, on stress, and regarding symptom alleviation in anxiety disorders in rodents and humans^27,63,74^. Bergamaschi et al.^75^ demonstrated that cannabidiol (CBD) reduced anxiety when given to subjects with an untreated social anxiety disorder before a public speech. Furthermore, Fusar-Poli et al.^76^ showed that emotional processing was modulated by THC and CBD in a way that THC increased the skin-conductance response during the presentation of fearful faces, whereas CBD administration led to a reduction of the same response. Focussing more on extinction learning, as the core of exposure therapies, based on data in healthy subjects, Rabinak et al.^36^ suggested a putative role for pre-treatment with THC to enhance extinction learning during exposure therapy. Another study found that consolidation of extinction learning was enhanced by cannabidiol administration post extinction learning^77^ and Mayo et al.^13^ found enhanced extinction recall in healthy humans pre-treated with FAAH-inhibitors.

Results from those previous studies together with the present main outcome that either higher baseline AEA levels or increases of AEA during extinction learning can add beneficially to this process suggest the consideration of pharmaceutical add-on treatment for psychotherapeutic interventions like exposure therapies.

As a prospective clinical proof of concept, patients with stress or anxiety disorders could be pre-treated with AEA-enhancing drugs to promote extinction learning^36^. Our data corroborate a potential role of the endocannabinoid AEA in the therapy of anxiety disorders^28,67^, but more research in humans is required to still better understand the underlying mechanisms. Nevertheless, our data underline the importance for future research to investigate circulating endocannabinoids in healthy humans and in patients with different anxiety disorders, especially before and after psychotherapy, before attempting to stimulate the ECS for beneficial effects.

## Supplementary information

Supplemental Material

Supplemental Figure 1
