# Supplementary material for: Fear extinction learning and anandamide: an fMRI study in healthy humans
Source: Transl Psychiatry. 2021 Mar 15;11:161. doi: 10.1038/s41398-020-01177-7 (PMC7961038; doi:10.1038/s41398-020-01177-7)
Supplement: Supplementary file 1 — Supplemental Material [file 41398_2020_1177_MOESM1_ESM.docx]

**Supplemental Information**

Materials and methods

*Participants*

Participants were native German-speaking, Caucasian, male students without past or current history of psychiatric or neurological disorder as assessed by a preclinical Structured Clinical Interview for DSM-IV, particularly no history of any drug or alcohol abuse/dependence. We chose solely male participants for the present study to minimise confounding factors in this first translational study, since previous studies have pointed out sex differences in extinction recall[^78^](#_ENREF_78), stress response circuitry adaptation[^79^](#_ENREF_79) and pain perception[^80^](#_ENREF_80). From an initial sample of n=55, data from four participants had to be excluded from final analyses due to technical failures (2), acute medical problems (1) and poor data quality (1).

Regarding lifetime exposure to cannabis, 27 of 55 participants had tried cannabis at least once previously, but no one reported regular use in the past. All participants confirmed complete abstinence from cannabis use in the two months before the experiment, and had negative THC-screening results. Further exclusion criteria were serious medical conditions, intake of any regular or acute medication (except for thyroid hormones). Participants were received financial compensation of 90 €.

*Temperature calibration of the thermode*

Before the start of the main experiment, the intensity of the unconditioned stimulus (US), an unpleasant thermal stimulation on the right shinbone, was determined. An fMRI-compatible ATS-thermode (30 x 30 mm, TSA-II, Medoc Advanced Medical Systems, Ramat Yishai, Israel) was applied to an area pre-treated with 0.5% capsaicin solution (Wörwag Pharma). The intensity of stimulation was individually determined by gradually increasing the temperature of the thermode until subjective unpleasantness of 6 out of 10 was reached on a 0 to 10 analogue scale, where 0 was "not unpleasant at all", and 10 was "the most imaginable state of pain”[^81^](#_ENREF_81). The number 6 on this scale indicated a thermal stimulation that was “clearly unpleasant, but still endurable and not painful”. This definition is in line with Lonsdorf et al. (2017), who report this level as commonly used in human fear conditioning setups[^6^](#_ENREF_6). Mean temperature over subjects was 46.59 °C, SD = 2.96 °C.

During the main experiment, temperature oscillated between +/- 1.6 °C on average. Ramping of the US started 2.5 s after CS^+^ onset at a rate of 8 °C/s to achieve maximal thermal stimulation at about 4 s after trial onset. Once reaching the peak temperature, the temperature decreased, again at a rate of 8 °C/s until reaching the baseline temperature of 32 °C, resulting in a trial length of about 6 s for thermal stimulation trials.

*Presentation set-up*

To program and present the trials, Presentation 14.8 (Neurobehavioral Systems Inc., Albany, CA) was used. Participants were able to see the stimuli presented on a 32” LCD display (NordicNeuroLab AS, Bergen, Norway) at a resolution of 1280 x 720 pixels, which is installed behind the MR scanner and was visible by means of a double-mirror mounted on top of the head coil.

*FAAH-genotyping*

DNA was extracted applying standard protocols of a commercial extraction kit (MagNA Pure 96 DNA and Viral NA Small Volume Kit) and the MagNA Pure 96 (Roche Diagnostics, Mannheim, Germany). Genotyping at FAAH C385A was performed via real-time quantitative polymerase chain reaction using melting curve detection analysis with Cobas z480 LightCyclers (Roche Diagnostics, Mannheim, Germany). The primers were obtained from TIB-Molbiol, Berlin, Germany.

*Mass spectrometry of anandamide*

Plasma levels of the endocannabinoid N-arachidonoylethanolamide (AEA) were assessed using mass spectrometry. For each participant, 7.5ml EDTA blood samples were directly centrifuged (10 min, 2000 g, 4°C) and 100 µL of blood plasma was directly pipetted and stored at -30°C for one week and then stored at -80°C for approximately 4 weeks.

Endocannabinoids were extracted from 100 µL human plasma by liquid-liquid-extraction (LLE). Plasma samples were allowed to thaw on ice for 90 min. Then, 250 µL of ethylacetate/n-hexane (9:1, v/v) and a 50 µL mixture of internal standards in acetonitrile, e.g. AEA-d4, 2-AG-d5, and AA-d8 were then added to the plasma samples. The concentration of internal standards in the spiking mixture was set so as to render a target concentration of the deuterated standards in the final sample extracts of 0,25 ng/ml for AEA-d4, 2,5 ng/ml for 2-AG-d5, 100 ng/ml for AA-d8, respectively[^82^](#_ENREF_82). Samples were vortexed for 30 sec and then centrifuged for 5 min at 4 °C and at 5080 g with a swing rotor. Following centrifugation, samples were kept at -20°C for 10 min for phase separation. The upper organic phase was recovered and pipetted in 96 deep-well plates and allowed to evaporate at 37 °C under a gentle stream of nitrogen. The extracts were then reconstituted in 50 µL acetonitrile/water (1:1, v/v). The solubilized extracts were then transferred in 96 microtiter plates for LC/MRM injection. LC/MRM conditions for analysis and quantification of eCBs were as described in[^82^](#_ENREF_82)^,^ [^83^](#_ENREF_83). Throughout the entire sample extraction procedure as well as in the LC/MRM autosampler samples were maintained at 4°C, and additionally all solvents and vials were pre-cooled at 4°C to prevent and/or standardize ex-vivo changes of eCBs levels across all samples. eCBs values were normalised to plasma volume. The data on 2-AG and AA are under evaluation and will be reported elsewhere (manuscript in preparation).

*MRI data acquisition*

For the estimation of task-related brain activity, the T2*‑weighted BOLD signal was measured using an echo-planar imaging (EPI) pulse sequence with the following parameters: repetition time (TR) = 2000 ms, echo time (TE) = 33 ms, flip angle = 90°, bandwidth = 2136 Hz/pixel, PAT factor = 2 (GRAPPA mode), field of view (FOV) = 220 mm, matrix size = 90 x 90, number of slices: 32, slice orientation: transversal, acquisition: ascending, slice thickness: 3.0 mm, interslice gap = 1.0 mm, voxel size = 2.44 mm x 2.44 mm x 4.00 mm.

A high resolution T1-weighted structural image was obtained by administering a 3D magnetisation prepared rapid acquisition gradient echo sequence TR = 2300 ms, TE = 2.98 ms, inversion time = 900 ms, flip angle = 9°, FOV = 256 mm, matrix size: 256 x 256, voxel volume = 1 mm^3^, slice orientation: sagittal; PAT factor = 2 (GRAPPA mode); scan time = 5.21 min.

*Image pre-processing of fMRI data*

The pre-processing and statistical analyses of the MRI data were performed using the software package Statistical Parametric Mapping (SPM) 12 (r6225; Wellcome Department of Cognitive Neurology, London, UK). Data from all participants met criteria for high quality and scan stability with minimum motion correction and were subsequently included in fMRI analyses (< 3 mm displacement in any one direction). The resting-state and task-related EPI series from all three days were first slice time corrected and spatially realigned to their respective mean EPI. Afterwards, the mean EPIs, and the T1 image were co-registered to the mean EPI of the first resting-state series. Next, for normalisation to Montreal Neurological Institute (MNI) space, the T1 image was segmented using SPM's "normalise" routine, and the resulting deformation field was applied to all pre-processed images. Subsequently, images had a voxel size of 2 x 2 x 2 mm^3^. Smoothing of the EPI images was performed using a Gaussian kernel with 8-mm full width at half maximum.

*Day 1 (Fear conditioning) Data analysis*

Per participant, pre-processed task-based fMRI data were modelled using a single sessions-separated general linear model. For the fear conditioning session (Day 1), both CS^+^ conditions were combined to form a regressor with onsets of CS^+a^ and CS^+b^ trials that were uncoupled with thermal stimulation. A second regressor modelled onsets of all CS^-^ trials, and a third regressor modelled all trials coupled with thermal stimulation. Trial onsets were convolved with the canonical hemodynamic response function (HRF) and its temporal derivative. Following model estimation, one-sided *t-*contrast images were computed for uncoupled CS^+^ and CS^-^ trials during fear conditioning. To test for significant effects of fear conditioning using this model, the contrast “CS^+^ minus CS^‑^“ was computed per each participant, and propagated to random effects group analysis.

*Day 2 (Fear extinction) Exponential decay function*

In order to estimate the decay in neural signalling during extinction learning, we made use of SPM's parametric modulation option. The first-level models therefore included not only two regressors representing the onsets of CS^+^ and CS^-^ trials, respectively, but also parametric modulation regressors, one for each condition. Modulation values were computed by entering the trial onset times (t) into the equation y = exp(-2t/tau) + C, with tau = total duration of the experiment divided by 8, and C = tau * (exp(‑total duration / tau) - 1) / total duration. Regressors were convolved with the canonical hemodynamic response function (HRF). The spatial realignment parameters were also added to the design matrix as regressors of no interest. Data were high-pass filtered (cut-off: 128 s) to remove low-frequency scanner drifts. An autoregression model of polynomial order 1 was used to account for temporally correlated residual errors.

Results

*Day 1 (Fear conditioning) Categorical effects of fear conditioning*

With the present paradigm, similar to previous meta-analyses^33,34^, we observed significant neuronal task-related activation during fear learning, that is, greater activation for CS^+^ relative to CS^-^ trials (Day 1) in the following cerebral networks: Bilateral anterior insula, bilateral rolandic operculum, bilateral striatum and thalamus, ventral tegmental area, middle cingulate cortex and supplementary motor area, and left middle frontal gyrus.

Supplemental Table 1. Brain regions with a significant effect of fear conditioning.

| *n* = 51. Contrast: “CS^+a/b^ minus CS^-^“. Significance: *p <* 0.001 at the voxel level and *p <* 0.05 (FWE-corrected) at the cluster level, corresponding to cluster sizes of at least 169 contiguous voxels. Abbreviations: L: left; R: right. | | | | | | |
| --- | --- | --- | --- | --- | --- | --- |
|  | Brain region | Number of voxels | Peak voxel (MNI space) | | | |
|  |  |  | x | y | z | *z*-score |
| R | Anterior insula | 3112 | 32 | 26 | 4 | 6.01 |
| L | Anterior insula |  | -34 | 28 | 0 | 5.78 |
| R | Ventral striatum |  | 12 | 6 | 4 | 5.68 |
| L | Ventral striatum |  | -8 | 10 | 0 | 5.17 |
| L | Ventral tegmental area |  | -2 | -20 | -12 | 5.07 |
|  | Cerebellum | 198 | 0 | -58 | -30 | 5.35 |
| L | Supplementary motor area | 794 | -2 | 8 | 60 | 4.63 |
| L | Middle cingulate cortex |  | -6 | 14 | 40 | 4.34 |
| L | Cerebellum | 415 | -10 | -80 | -30 | 4.62 |
| R | Cerebellum | 348 | 12 | -76 | -28 | 4.54 |
| L | Middle frontal gyrus | 194 | -30 | 48 | 2 | 4.38 |

*Anxiety ratings*

On all days after the experiment, and on Day 2 and Day 3 additionally before the experiment, participants were asked to rate “*How afraid are you of the stimulus coupled to this symbol?”* on a scale from 0-10 (*not at all – very much*). The summarised results are presented in *Supplemental* *Figure* *1*. Significant differences from paired t-tests, were found on Day 1 between CS^+a^ (M = 7.54, SD = 1.73) and CS^-^ (M = 0.76, SD = 1.56) (t(50) = 18.33, *p <* 0.001) and between CS^+b^ (M = 7.42, SD = 1.90) and CS^-^ (t(50) = 18.02, *p <* 0.001), supporting successful fear conditioning.

**Supplemental Figure Legend**

Supplemental Figure 1. Anxiety ratings for fear conditioning on Day 1. Error bars denote standard error of the mean. Asterisks represent significant group differences (* p < 0.05; *** p < 0.001). Abbreviation: a.u.: arbitrary unit.

Supplemental References

**6. Lonsdorf TB, *et al.* Don't fear 'fear conditioning': Methodological considerations for the design and analysis of studies on human fear acquisition, extinction, and return of fear. *Neurosci Biobehav Rev* 2017; 77: 247-285.**

**33. Fullana MA, *et al.* Fear extinction in the human brain: A meta-analysis of fMRI studies in healthy participants. *Neurosci Biobehav Rev* 2018; 88: 16-25.**

**34. Fullana MA, *et al.* Neural signatures of human fear conditioning: An updated and extended meta-analysis of fMRI studies. *Mol Psychiatry* 2016; 21(4): 500-508.**

78. Shvil E, *et al.* Sex differences in extinction recall in posttraumatic stress disorder: a pilot fMRI study. *Neurobiol Learn Mem* 2014; 113: 101-108.

79. Goldstein JM, Jerram M, Abbs B, Whitfield-Gabrieli S, Makris N. Sex differences in stress response circuitry activation dependent on female hormonal cycle. *The Journal of neuroscience : the official journal of the Society for Neuroscience* 2010; **30**(2)**:** 431-438.

80. Wiesenfeld-Hallin Z. Sex differences in pain perception. *Gender medicine official journal of the Partnership for Gender-Specific Medicine at Columbia University* 2005; **2**(3)**:** 137-145.

81. Attar CH, Finckh B, Buchel C. The Influence of Serotonin on Fear Learning. *PLoS ONE* 2012; **7**(8).

82. Bindila L, Lutz B. Extraction and Simultaneous Quantification of Endocannabinoids and Endocannabinoid-Like Lipids in Biological Tissues. *Methods Mol Biol* 2016; **1412:** 9-18.

83. Post JM, *et al.* Antiepileptogenic Effect of Subchronic Palmitoylethanolamide Treatment in a Mouse Model of Acute Epilepsy. *Front Mol Neurosci* 2018; **11:** 67
