# Supplementary figures and images for: Fear extinction learning and anandamide: an fMRI study in healthy humans
Source: Transl Psychiatry. 2021 Mar 15;11:161. doi: 10.1038/s41398-020-01177-7 (PMC7961038; doi:10.1038/s41398-020-01177-7)

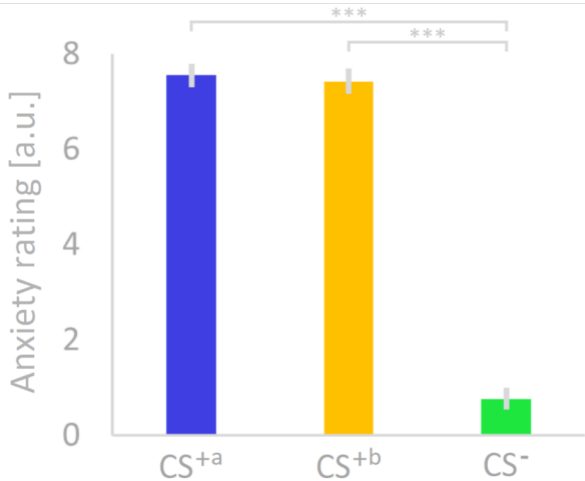

Supplement: Supplementary file 2 — Supplemental Figure 1 [file 41398_2020_1177_MOESM2_ESM.pdf]
